# A systematic review of vital events tracking by community health agents

**DOI:** 10.1080/16549716.2019.1597452

**Published:** 2019-06-10

**Authors:** Erin K. Nichols, Nina W. Ragunanthan, Braveen Ragunanthan, Hermon Gebrehiwet, Karim Kamara

**Affiliations:** a National Center for Health Statistics, Centers for Disease Control and Prevention, Hyattsville, MD, USA; b Department of Health and Human Services, United States Public Health Service, Washington, DC, USA; c Department of Obstetrics, Gynecology, and Reproductive Sciences, University of Pittsburgh Medical Center Magee-Womens Hospital, Pittsburgh, PA, USA; d Department of Pediatrics, University of Pittsburgh Medical Center Children’s Hospital of Pittsburgh, Pittsburgh, PA, USA; e Health Sciences Program, Argosy University, Arlington, VA, USA; f School of Health Professions, Shenandoah University, Winchester, VA, USA

**Keywords:** Vital statistics, mortality surveillance, verbal autopsy, community health workers, civil registration

## Abstract

**Background**: Efforts to improve national civil registration and vital statistics (CRVS) systems are focusing on transforming traditionally passive systems into active systems that have the ability to reach the household level. While community health agents remain at the core of many birth and death reporting efforts, previous literature has not explored elements for their successful integration into CRVS efforts.

**Objective**: To inform future efforts to improve CRVS systems, we conducted a systematic review of literature to understand and describe the design features, resulting data quality, and factors impacting the performance of community health agents involved in tracking vital events.

**Methods**: We reviewed 393 articles; reviewers extracted key information from 58 articles meeting the eligibility criteria: collection of birth and/or death information outside of a clinic environment by a community agent. Reviewers recorded information in an Excel database on various program aspects, and results were summarized into key themes and topic areas.

**Results**: The majority of articles described work in rural areas of Africa or South-East Asia. Nearly all articles (86%) cited some form of household visitation by community health agents. Only one article described a process in which vital events tracking activities were linked to official vital events registers. Other factors commonly described included program costs, relationship of community agents to community, and use of mobile devices. About 1/3 of articles reported quantitative information on performance and quality of vital events data tracked; various methods were described for measuring completeness of reporting, which varied greatly across articles.

**Conclusions**: The multitude of articles on this topic attests to the availability of community health agents to track vital events. Creating a programmatic norm of integrating with CRVS systems the vital events information collected from existing community health programs has the potential to provide governments with information essential for public health decision-making.

## Background

While the registration of all births and deaths is a fundamental obligation of government, this obligation is not met in most low-income countries. This failure denies children a basic human right – proof of their identity – and deprives government of essential information to address population health, education, and other important needs [1]. Among these needs, accurate and timely vital statistics data, including cause of death information, are critical to inform public health decision-making. This critical information is particularly lacking in countries in sub-Saharan Africa, where the proportion of births registered averages only about 56% [2].Furthermore, only 7 countries in Africa have complete death registration systems [3], and only 4 countries report to the World Health Organization cause of death statistics of any level of quality [4]. This ‘scandal of invisibility’ [1] has created an information paradox, with information lacking where it is most needed.

**Figure 1.**
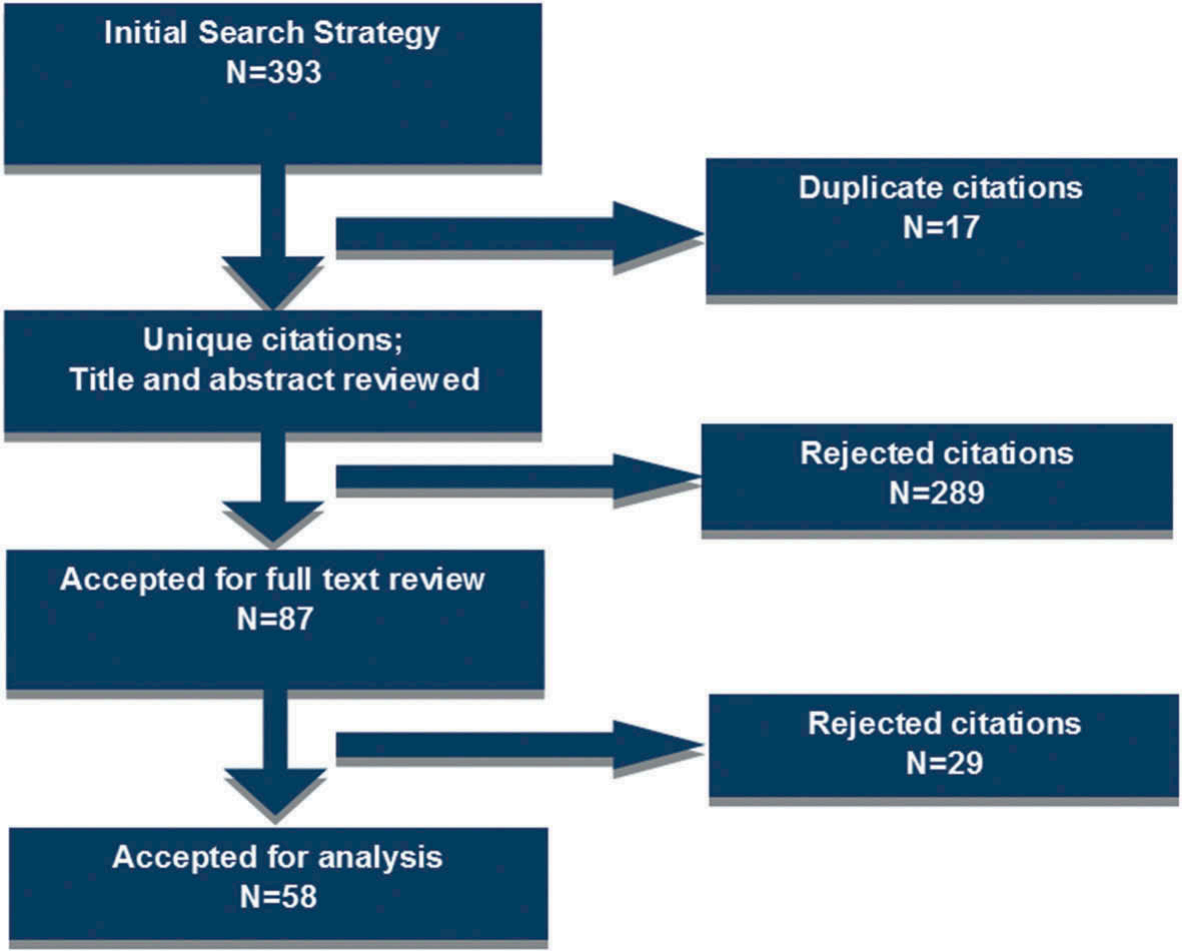
Flow Diagram of Literature Search Strategy.

**Figure 2.**
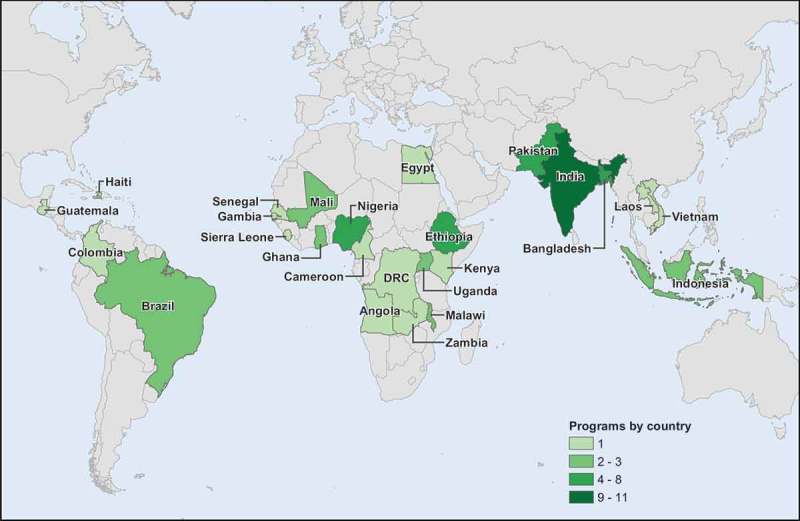
Countries Represented in Literature Review.

Well-functioning national civil registration and vital statistics (CRVS) systems are best placed to provide high-quality vital statistics information. However, outdated or insufficient legislation, inaccessible registration centers, unreliable services, a lack of awareness of the value of birth and death certificates, and a lack of incentives or services requiring use of certificates have prevented the establishment of strong CRVS systems in low-resource settings [1].

Despite the many challenges, recent developments have set the stage for a renewed effort to improve national CRVS systems. The measurement of Millennium Development Goals 4 (reduce child mortality), 5 (improve maternal health), and 6 (combat HIV/AIDS, malaria, and other diseases) and subsequently multiple Sustainable Development Goals has placed a sense of urgency on countries to improve the availability of useful vital statistics data. Numerous international organizations have recognized the need for such information in low-income countries, and regional platforms have been established to support system development, including the United Nations Economic Commission for Africa (UNECA) Africa Programme on Accelerated Improvement of CRVS (APAI-CRVS) [5] and the United Nations Economic and Social Commission for Asia and the Pacific (UNESCAP) Get Everyone in the Picture [6] platform to promote CRVS. Furthermore, in some countries, economic development has led to the advancement of effective government infrastructure that can support CRVS system strengthening. Approaches such as sample registration systems and the use of mobile technology have been shown to work in a variety of settings and can support CRVS system strengthening.

A community agent system, including Community Health Worker (CHW) platforms, can create a key linkage in transforming traditionally passive systems (i.e. waiting for individuals to report to the system) of vital registration into active systems (i.e. agents connect to the individuals/family to report events) that have the ability to reach the household level even in the most remote areas. However, while community agents remain at the core of many birth and death reporting efforts, previous literature has not explored the key elements for their successful integration into CRVS efforts. As regional platforms are simultaneously gaining momentum for the expansion of programs, including the One Million CHW Campaign in sub-Saharan Africa [7], it is important to consider how common interests within community agent/CHW and CRVS improvement efforts can be leveraged for maximum impact.

In this paper, we provide results of a global systematic review of published literature to understand and describe programs where community agents have been deployed to track community births and deaths. We describe the program design features, resulting data quality, and factors impacting performance related to supporting the official notification and registration of tracked events within a national CRVS system. Results suggest the potential that CHW programs can offer in support of CRVS improvement, yet bring to light key issues for consideration in fully leveraging their capacity.

## Methods

### Search strategy

We conducted a systematic review of literature published in English between the years of 1946 to 2016 using the PRISMA statement as guidance [8]. A written protocol is not available for this review; see Table S1 for the PRISMA checklist. The search included MEDLINE, EMBASE, and Global Health databases. To capture multidisciplinary evidence the search was extended to a sociological database; however, the search did not result in any relevant articles. The strategy for the search, including search terms, is summarized in Table 1 below, with results from an initial search conducted in 2014.

**Table 1.** Literature search strategy used in databases.

Search order	Search terms used
1	Community Health Workers/
2	(community health worker* or frontline health worker* or outreach worker* or community health education worker* or lay health worker* or promotora* or village health worker* or volunteer health worker* or community health distributor* or community health surveyor* or community health assistant* or community health promoter* or community health agent* or rural health auxiliar* or rural health worker* or birth attendant* or skilled attendant* or health promoter* or community cadre*).tw.
3	1 or 2
4	(birth certificate* or death certificate* or vital statistics or vital registr*).mp.
5	((regist* or record* or report* or collect*) adj6 (birth* or death* or mortality)).tw.
6	verbal autops*.tw.
7	4 or 5 or 6
8	3 and 7
9	8 not (exp north america/or exp australia/or exp new zealand/or exp europe/)

The term ‘Midwives’ was deleted from the search strategy because it returned about 185 unique items that were all irrelevant.

The initial search was conducted in 2014 for articles published through 2013; updated searches were conducted for articles published through 2016, and an additional five articles known to the authors were also included. A total of 393 articles were listed in the initial search; after removing 17 duplicates, 376 articles were reviewed in the initial screening process (see Figure 1).

In the initial screening process, article titles and abstracts were evaluated based on the following eligibility criteria:

1. Mention of data collection by community agent, including community health workers (CHWs) and traditional birth attendants (TBAs);
2. Any form of reporting, recording, and/or registering of births and/or deaths and information related to these events, including verbal autopsy and cause of death; and
3. Information is collected outside of a clinic environment; i.e. the community agent serves as an outreach worker visiting the home or other location to collect information; information collection is ‘active,’ where the community agent seeks the information, not ‘passive,’ requiring the family to come to the facility to report the information.

Five reviewers, including the principal investigator, completed the screening process to determine which articles would be included in a full text review. Inclusion criteria applied in the full article review were based on the same eligibility criteria as was applied to the title and abstract review step; articles were excluded if they were not available in English full text, or if they did not meet the eligibility criteria described above. Reviewers documented their results in an Excel database. The screening process resulted in 87 articles eligible for the full text review. In the second phase, four reviewers extracted key information from the articles and recorded the information in an Excel database. Extracted information included the following information: reference information; demographics; timeline; nature of project; study/project design; program background; operational snapshot; results/outcome of study/program; how data are used; challenges and limitations; and opportunities/benefits.

After the full text review was completed, 29 articles were determined to be irrelevant and were removed from further analysis; 58 articles on 44 unique studies or projects were considered relevant for analysis (for the purposes of this review, all articles were analyzed independently). The principal investigator reviewed all results to confirm article selection. Results were summarized into key themes and topic areas related to the considerations, supportive factors, and barriers for involving community health agents in tracking vital events; frequencies of key factors are reported.

At the start of analysis, the varying terms used to describe community agents were organized into four categories, as described in Table 2. Each cadre of worker was defined by three criteria: payment; health focus; and location of service provision.

**Table 2.** Categorization of community agent terminology.

Title	Description	Other titles used
Community health workers (CHWs)	Minimal to no payment; broad health focus; outreach-based service	*Anaganwadi* worker, community health promoters, community health agents, *Kaders*, village health workers (VHWs), *Promotoras*, health surveillance assistants, community health development agents, and *Relais communautaires*
Health extension workers (HEWs)	Paid; broad health focus; facility-based service	Health Assistants (HA), Family Welfare Assistants (FWA), lady health workers, multi-purpose worker
Surveillance agents	Paid; health focus; outreach-based service	Disease surveillance officers
Birth attendants	Payment varies; reproductive/health focus; outreach and facility-based service	Traditional birth attendants (TBAs)

## Results

### Scope of review

These articles were published between 1983 and 2016, with a median year of 2009. Articles represent studies or projects from 24 countries in Africa (n = 29), the Americas (n = 4), Eastern Mediterranean (n = 5), and South-East Asia (n = 22) (See Figure 2). A total of 47 articles described projects in rural areas, five described projects in urban areas, and six articles included both urban and rural populations. Regarding the types of studies, one study was experimental, and 13 were quasi-experimental (five with concurrent control group, and eight without concurrent control group). The rest were non-experimental.

Regarding program type, studies or projects based on routine government programs were described in 12 articles, on routine government programs enhanced in some way through external technical and/or financial support in 20 articles, on donor-sponsored programs in six articles, and term-limited research studies in 19 articles; one article presented a general commentary on methodological issues. Vital events tracking was conducted by CHWs in 41 articles, health extension workers (HEWs) in nine articles, surveillance agents in two articles, and by birth attendants in 10 articles (note: some articles described multiple cadres). Other types of community agents, including community leaders, village heads, religious leaders, and school children, contributed to vital events tracking in eight articles. Community agents collected information on births in 46 articles and deaths in 55 articles (age groups shown in Table S2). Cause of death was documented in some way in 38 articles. Use of verbal autopsy or some attempt to get cause of death through a household interview was indicated in 29 articles, with the interview being conducted by birth attendants (n = 1); CHWs or VHWs (n = 7); midlevel health personnel, including HEWs, health assistants, surveillance agents, nurses, or midwives (n = 8); by study personnel or field supervisors (n = 12); or by a physician (n = 2). See Tables 3 and S2.

**Table 3.** Summary of Scope of Review.

Category		Article n (%)^a^
Location	Africa	29 (48.3)
	Americas	4 (6.7)
	Eastern Mediterranean	5 (8.3)
	South-East Asia	22 (36.7)
Urbanicity	Urban	5 (8.6)
	Rural	47 (81.0)
	Multiple sites or national	6 (10.4)
Type of program	Routine program	6 (10.3)
	Enhanced routine program	26 (44.8)
	Donor-sponsored program	2 (3.4)
	Research study	23 (39.7)
	General commentary	1 (1.7)
Type of community health agent	Community health workers	41 (58.6)
	Health extension workers	9 (12.8)
	Surveillance agents	2 (2.9)
	Birth attendants	10 (14.3)
	Other	8 (11.4)
Vital event collected	Births	46 (79.3)
	Deaths	55 (94.8)
	Cause of death	38 (65.5)
	Verbal autopsy	29 (50.0)

^a^ Where totals do not sum to 58, articles mentioned multiple categories.

### Program design

This section summarizes various aspects of program design including: health area and population of focus; program duration; reporting practices; selection of community agents; cost information; use of mobile devices; and involvement of non-health related community informants in reporting vital events. Results are summarized in Table S2.

#### Population of focus

The search criteria returned articles focusing on a variety of populations and health program outcomes. For articles focusing on health program outcomes, the population of focus was women of reproductive age or children for the vast majority of articles – specifically: women of reproductive age for 25 articles, pregnant women for 24 articles, children (0–4 yrs, under 5, 0–14 years, newborns, infants) for 23 articles; one article focused on adults 50 years and older. Eight articles focused on training or performance outcomes or perspectives of a cadre of health professionals. Fifty-four articles described results from programs or studies that had concluded within a specified time range, while four articles described programs that appeared to continue past the time of publication.

#### Program structure and cost

Nearly all articles (n = 50) described household visitation as an active method used by community health agents to provide services and/or collect vital events information. Pregnancy surveillance as a method to track birth outcomes was reported by 21 articles. While such information was not available from all articles, 22 articles indicated that the community health agents were selected from within community. Cost information was provided on salary or payment, including transport allowances or phone credits for nine articles, accessories for community health agents for three articles, and payment for each birth or death recorded was reported by one article. Community health agents were reported as serving as unpaid volunteers in nine articles, and as working in hospitals in one article. Overall program cost was provided by 10 articles, and the cost of the program cited in terms of cost per outcome (per-unit cost) was provided by four articles.

#### Use of mobile devices

Starting in 2009, 10 articles mentioned use of mobile devices to support the work of community agents and reporting births and deaths. In a multi-country study in South-East Asia, mobile devices were used for delivery notification purposes [9]. In Nigeria, *Resident Association Chairmen*, non-health focused community leaders responsible for recording births and deaths and monitoring population movement, reported events through texts on mobile phones [10]. In a program to report postpartum hemorrhage in rural Ghana, birth attendants were trained to use mobile phones to report maternal and neonatal deaths via short message services (SMS) text messages [11]. In Uganda, CHWs used mobile phones to report births and deaths and as a support tool for community case management of childhood illness [12]. In Senegal, the Millennium Villages Project tested the ChildCount+ platform which uses SMS texts or smartphone applications to facilitate and coordinate activities of CHWs, including vital events data collection. This program also tested use of mobile phones by specially trained field workers to conduct verbal autopsy [13]. Ohemeng-Dapaah describe testing also conducted by the Millennium Villages Project on use of a Java-enabled mobile phone by CHWs to report data, though that was not the focus of the article included in this review [14]. Joos et al. used a cluster-randomized intervention among CHWs (*Health Surveillance Assistants)* in Malawi to test whether supportive SMS texts could improve reporting of pregnancies and pregnancy outcomes; they concluded that mHealth applications have the potential to improve the tracking and data quality of pregnancies and pregnancy outcomes, particularly in low resource settings [15]. Results of this testing are further described by Silva et al., as part of a three-country assessment of community-based methods to support real-time measurement of mortality [16]. In a quasi-experimental study in rural Lao People’s Democratic Republic, Xeuatvongsa et al. studied the impact of the provision of a mobile phone and phone credits, along with training and outreach per diem, on timeliness of birth notification by village health volunteers to healthcare workers and subsequent provision of hepatitis B birth dose; they concluded that the provision of phones and phone credits might be one important factor for increasing immunization coverage [17].

#### Collaboration with other reporting systems

Six articles described involvement of non-health related community informants in reporting vital events. Of these, two articles described use of the MADE-IN/MADE-FOR (Maternal Deaths from Informants/Maternal Death Follow on Review) approach to using village informant networks to capture maternal death cases [18,19]. Qomariyah et al. describe using this approach in Indonesia, where lists of maternal deaths prepared by a network of village heads were compared to lists prepared by health volunteers using capture-recapture analysis [19]. This approach was also tested in Pakistan, where village imams, lady health workers, elected lady councilors, and marriage registrars, participated in networks to develop lists of maternal deaths [18]. Two additional articles mentioned use of religious leaders or institutions to help report events. One program in Ethiopia trained priests to work alongside community health workers to conduct sentinel surveillance; priests were responsible for documenting births and female deaths, while CHWs visited the churches six days a week to collect the records [20]. In another program in Nigeria, community health promoters (CHPs) visited churches and mosques to collect birth and death information for maternal and perinatal deaths [10]. In this same project, CHPs also visited *Resident Association Chairmen* who were responsible for recording births and deaths and monitoring population movement in and out of the project area. Similarly in a program in India, community gatekeepers called *Chowkidars*, who are responsible for recording births and deaths from information reported by traditional birth attendants and village friends, were used to compare reporting by TBAs in the same communities [21]. Finally, a program in Zambia used students to notify CHWs when births or deaths occurred and to record data [22].

Only one article described a process in which vital events tracking activities were linked to official vital events registers [20]. Four additional articles [15,17,21,23] describe use or testing of birth/death registration forms or systems, or an outcome of interest being increased birth notification, but these articles included no indication of actual linkage to official civil registration processes.

### Quality of data tracked by community health agents

As this review sought to capture and summarize methodological information about birth and death reporting, articles were not judged for inclusion on the basis of a rigorous assessment of quality of resulting outcomes. However, among the articles reviewed, 18 contained quantitative information on the performance and quality of vital events data tracked by community agents. In 11 of these articles, the focus of the article was on the quality of vital events data tracked by community agents, while for the remaining 7 articles, quality was reviewed as a subcomponent of a larger project described in the article. Among the 18 articles containing quantitative information, only three reported data from routine surveillance systems; most were reporting data from research programs or ‘enhanced routine’ programs, in which existing CRVS systems had been modified (see Table S2.) Six of the 18 studies were quasi-experimental or experimental in design, and the rest were non-experimental (retrospective, prospective cohort, or cross-sectional studies).

#### Completeness of vital events reported

Completeness of vital events reported by community agents was evaluated against an independent household audit or survey in six projects reviewed. Household validation in the Real-time Monitoring of Under-five Mortality (RMM) initiative included a full birth or full pregnancy history from women of reproductive age to determine annualized expected births, under-five deaths, and under-five mortality rates. Completeness of birth and under-five death reporting was 30.1% and 21.7% by HEWs in Ethiopia [16,24,25], 65.9% and 50.6% by Health Surveillance Assistants (HSAs) in Malawi, and 90.3% and 90.8% by community health volunteers (*Relais*) in Mali [16] respectively. In India, Village Health Workers (VHWs) reported an estimated 98% of births and childhood deaths as compared to a house-to-house survey every six months by a VHW from another village to detect missed births and deaths; the Chandrasekar-Deming method was used to estimate the 2% missed events [26]. In another project in Uttar Pradesh, India, community health workers (*Anganwadi* workers) reported 63.4% of births and 86.1% of all deaths as compared to a household visit by a middle level supervisor [27]. In Uganda, CHWs reported 18% fewer child deaths as compared to an annual household audit [28].

Completeness of vital events reported was evaluated by a comparison to official or expected figures based on non-household data sources in four projects reviewed. The Real-time Monitoring of Under-five Mortality (RMM) initiative also measured completeness of birth and death reporting against village health registers in Malawi, where HSAs reported 87% of births and 89% of deaths, and against family folders maintained at local health posts in Ethiopia, where HEWs reported 93% of births and 91% of deaths [16]. In Vietnam, the accuracy of neonatal mortality rates based on reports through group interviews with VHWs was measured to be 16 per 1,000 as compared to an official reported rate of 4.2 per 1,000 [29]. In rural Ethiopia, HEWs reported 81.4% of expected births through household visits, compared to the annual crude birth rate estimate; the same study showed a maternal mortality ratio (MMR) of 489 per 100,000 live births, which was within the 95% confidence interval of the expected MMR of 531 (95% CI: 413–669) [30]. Finally, in Indonesia, Village Surveillance Agents (VSAs) reported births and deaths with 75% completeness compared to annual vital statistics data [31].

Two projects measured reporting coverage using a comparison of two community networks, through the MADE-IN/MADE-FOR approach. Using capture-recapture estimation, the Lady Health Workers reported 73% of pregnancy-related deaths in rural Pakistan, while religious leaders reported 49% [18]. In Indonesia, health volunteers reported 71% of pregnancy related deaths, while heads of neighborhood units reported 85%; accuracy of reporting was higher for health volunteers, more recent deaths, and rural deaths [19].

When measuring agreement on cause of death classification as reported by health workers versus hospital-generated information in Cameroon, 70% agreement was reported with four Global Burden of Disease death categories, while 80% agreement was reported between health worker classification of death and hospital-generated cause of death [32].

A study in northern India compared birth and death reporting completeness among traditional birth attendants (TBAs), Multipurpose Health Workers (MPWs), and *Chowkidars* (village watchmen). Coverage of births, stillbirths, and neonatal death reports by each cadre were compared to a total number of reports calculated by study personnel and showed 95.9%, 95.8%, and 95.8% coverage for TBAs; 75.9%, 62.5%, and 77.5% coverage for MPWs; and 24.5%, 12.5%, and 5.0% coverage for *Chowkidars* respectively [21].

Finally, the Real-time Monitoring of Under-five Mortality (RMM) initiative in Malawi also measured matched pregnancy documentation and outcome, including live birth, stillbirth, maternal deaths, and neonatal deaths, as reported by HSAs and as confirmed by a data editor via call to the reporting HSA. HSAs who did not reside in their catchment area were 28% more likely to match pregnancies than HSAs who did reside in their catchment area; HSAs reported up to 46% more matched pregnancies after receiving one-way short message services (SMS) sent with motivational content [15].

#### Methods of validating verbal autopsy data

Two articles reported methods of validating verbal autopsy data. In India, field supervisors visited VHWs every 15 days to verify births and childhood deaths using verbal autopsy [26]. In a multi-site study including the Democratic Republic of the Congo, Guatemala, Pakistan, and Zambia, concordance of responses to verbal autopsy questions was measured between the respondent and birth attendants in a sample of 234 deliveries for which there was concordance between the mother and the birth attendant as to the time of the death. For early neonatal deaths, concordance across all questions was 94%; concordance on any given question was not less than 80%, and it was at least 95% for more than half the questions on maternal medical history, birth attendance and neonate characteristics; more than 80% of questions had a sensitivity of at least 80% and a specificity of at least 90%. For stillbirths, concordance across all questions was 93%; concordance was 95% or greater for more than half of the questions on birth attendance, site of delivery and stillborn characteristics; more than 60% of the questions had a sensitivity of at least 80%, and more than 80% had a specificity of at least 90%. Overall, cause of death established through verbal autopsy were similar, regardless of respondent [33].

#### Other data quality measures

Other data quality measures were also reported. The Real-time Monitoring of Under-five Mortality (RMM) initiative measured the accuracy of crude birth (CBR) and death rate (CDR) calculations based on the RMM method compared to estimates derived from household-based full birth histories (FBHs). The community health volunteers (*Relais*) in Mali performed best with a CBR and under five mortality rate (U5MR) accuracy above 90% and accuracies above 100% for infant (IMR) and neonatal mortality rates (NMR). The Health Surveillance Assistants in Malawi reached accuracies around 65%-80% for all measures, while the HEWs in Ethiopia showed underreporting with CBR and U5MR accuracies under 50%, an IMR accuracy around 70%, and an NMR accuracy around 90% [16]. The RMM initiative also used data quality metrics traditionally used in demography. Comparing reports from community agents (RMM) to Full Pregnancy Histories, sex ratios at birth were about 95 and 101 in Malawi, about 112 and 99 in Mali, and both about 100 in Ethiopia, respectively [16].

The RMM initiative also measured timeliness of community health agents reporting of monthly data on pregnancies, births, and deaths. CHWs submitted monthly vital event reports for over 95% of catchment areas in Ethiopia and Malawi, and for 100% of catchment areas in Mali [16,24,34].

A study from rural Nigeria reported on the evaluation of record keeping practices for monitoring and evaluation and follow up/home visits based on a survey of VHWs and TBAs. Approximately 96% of respondents reported keeping records of their health activities; the same percentage reported forwarding their records. Nearly 90% felt record-keeping was easy; only about 6% of respondents said record keeping was difficult, while about 9% felt it demanded too much time [35,36].

A final data quality measure reported in this review was from a Ugandan study measuring continuity of CHW activity. After three years, 86.3% of CHWs were reported as still active, with more than 95% reported monthly attendance; however, only 24% of households reported that a CHW had visited their home within the past month [28].

## Discussion

This review summarizes experiences of community agents in tracking data on births and deaths, as described in 58 published articles. With 85% of the articles representing activities in Africa or South-East Asia, where key information on births and especially deaths is most lacking, this review shows that births and deaths are, in fact, being reported by a multitude of health programs. However, only one article described a process in which vital events tracking activities were linked to official vital events registers [20]. Given the number of health programs tracking vital events, it could be expected that efforts to officially compile, notify, register, and analyze the vital events reported through health programs could improve coverage of official vital statistics, therefore improving the value of vital statistics reports to countries for development, planning, and evaluation purposes.

### Factors impacting performance

Findings vary across articles, making it difficult to provide generalized guidelines, and some processes will vary according to local customs, beliefs, and conditions. However, synthesis of the data presented point to a number of factors that could impact the performance of community agents in tracking vital events data. First of all, that 50 of 58 (86%) articles describe approaches involving household visitation of community agents speaks to the important role of that it plays in the active tracking of vital events. For example, Perry et al. examine key aspects of routine systematic home visitation (RSHV) by CHWs and note that RSHV facilitates contact with all homes in a catchment area, enables a census to be taken and regularly updated, and supports prospective reporting and notification of vital events. They further note that RSHV aids in identifying and locating at risk individuals and populations and reduces geographic, socioeconomic, informational, and trust-related barriers to accessing basic health services. While they note that the RSHV approach requires close supervision and some type of incentive to encourage CHWs to continue their tasks, minimally paid and trained community agents can keep program costs feasible [37].

Upon qualitative review of the 58 articles, five key themes emerged as impacting the success of vital events registration: administrative affairs; community dynamics; relationship building; cultural considerations; and methodology. Each of these will be discussed in turn. Regarding administrative challenges to undertaking vital events registration, many articles mentioned geographic factors as barriers. These geographic factors included low population density, large geographic catchment areas, increasing distance from a HEW station, and long distances between houses [16,24,30,31,33,38,39]. Migration of the community was also noted as a challenge in areas in which there is a nomadic population or in cases in which the effect of migration within the community is not clear [24,34]. Another administrative issue that emerged was training and developing CHW staff. Some articles mentioned frequent turnover of staff as a challenge [16,24]. Some articles proposed solutions to the barriers of retention, including providing adequate materials [17,21,36] or providing remuneration [11,22,40]. Other articles mentioned difficulty in providing frequent and routine supervision of the health workers [24,34]. Many articles also recommended providing continuous training or frequent refresher training in order to ensure adequate capabilities of their CHWs [28,35,36,41].

The second theme that emerged was community. Many articles reported that their health workers were members of the community in which they worked, which was noted to promote trust, acceptance, and community engagement [16,32,39,42,43]. It was also noted as beneficial to have health workers that speak the same language and dialect as the community members [31]. However, some articles noted that the acceptance of their health workers was a challenge, such as in situations where a different community leader, such as a traditional healer, was held in higher regard [44]. It was also noted that community members were at times reluctant to disclose health information to CHWs if they perceived them as strangers [45]. Acceptance of the health workers is likely a key factor in successful implementation of community-based vital events reporting.

Similarly, fostering relationships between the CHWs and other workers emerged as a third key theme. Some articles emphasized the importance of cooperation and collaboration between local authorities, researchers, and CHWs [39,46]. One article noted that their CHWs had tensions with elected officials due to concerns over favoritism, issues of political visibility, and questions regarding authority to organize community events [22]. One article noted that to foster relationships among the CHWs, it was important to have those in leadership avoid a hierarchical or autocratic organizational structure, in order to provide dignity and respect to the CHWs [26].

Cultural considerations were the fourth theme noted to impact CRVS programs. Some articles noted that gender bias may have compromised the ability to obtain accurate birth registries, as female births were less likely to be reported than male births [16,21,27]. Similarly, births of infants born with low birth weight or congenital anomalies may be less likely to be reported [21]. Regarding documentation of deaths, one article noted that it was easier to document deaths than it was to document births because there were more community rituals and rites associated with deaths that marked the occasion [27]. However, other articles noted that it was more challenging to document deaths due to emotional and social sensitivities surrounding deaths in a household, as families were likely to withhold sensitive or embarrassing information – especially if the death was related to abortion, miscarriage, or suicide [14,18,19,30,47]. Similarly, in one article it was noted that mothers did not want to discuss a deceased child at all because of cultural beliefs [33].

The last theme that emerged across articles was a discussion of strengths and weakness of different methods of collecting vital events information. Many articles focused on the importance of active collection of information at the household level through regular household visits to capture people who do not seek healthcare in a healthcare setting (see Table S2). Other articles mentioned that using a variety of sources, including home visitations but also written records or reports from existing community-based networks, was important for capturing events [18,48]. Several articles noted challenges in accurate reporting of deaths due to recall bias, limitations in lay-reporting, under-recognition of underlying causes of death such as malnutrition or abortion, and unclear classification of overlapping causes of death such as AIDS and tuberculosis [16,19,21,23,26,34,49,50]. Additionally, two articles noted that there may be underreporting due to conflict of interest when CHWs or TBAs who are tasked with reducing adverse events such as maternal or neonatal deaths are asked to report on those events [11,24]. Finally, regarding project/study methods, mobile phones and SMS messaging were used to assist in timely reporting of births and deaths in several articles [11–17,25,38]. One article noted that mobile phone use increased the reporting of previously hard-to-capture events such as miscarriages, abortions, and stillbirths [15].

### Limitations

This review is limited to published literature and is dependent on the information selected for presentation in the articles. With regard to the publication bias, the review is restricted to information available from settings where programs to improve birth and death outcomes have been implemented and published. Though the age of some articles may diminish their relevance, the basic methods for reporting births and deaths have not changed during the time period of the review. While many programs may deploy vital events tracking as part of their efforts, programs were only included in this review if the relevant article included sufficient detail on the vital events tracking processes. A related issue is the inconsistency of terminology used for and detail provided on the various community agents and types of health workers described in this collection of articles. By categorizing the job-related details noted in Table 2, we attempted to validate and consistently apply the inclusion criteria for community agent, but our review was again limited to the information provided in the papers. Another issue related to terminology is the inconsistent use of language for describing tracking, reporting, notification, and registration processes, particularly with regard to the official civil registration processes of a country. Again, we are restricted to the details presented in the articles, and many cases remain unclear if these terms are used with reference to project-specific processes for reporting information on birth and death, or if they are in fact related to official notification and registration processes where birth and death information is submitted to civil registration authorities. Of particular challenge is the case where birth and death events may be officially reported within a national health system, but it is not explicitly confirmed if the events have been reported to or registered by civil registration authorities, from which a birth or death certificate could be issued.

While the review includes information about verbal autopsy or other such community-based processes to get cause of death information when these processes were conducted by community agents, many articles described such processes as conducted by a supervisor or health worker with a higher level of training, often as part of a validation visit to the household following report of a death by the community agent; these activities are not described in our review. Finally, articles selected for review mostly focus on health programs for children and women of reproductive age; limited information is available for all populations, particularly with regard to tracking all deaths.

## Conclusions

As global investment in strengthening CRVS systems gains momentum, information on active means for collecting birth and death information from the community are of increasing interest. The multitude of articles on this topic attests to the availability of community health agents to serve in this capacity, and this review attempts to highlight key considerations, supportive factors, and barriers to success. Creating a programmatic norm of integrating with CRVS systems the vital events information collected from existing community health programs could increase the coverage of officially reported vital events, providing governments with information essential for public health decision-making. Further exploration of how the CRVS community can best leverage community health programs conducting household visitation to include birth and death notification processes could prove useful. As the majority of programs or evaluation/monitoring systems applied are dependent on donor funding, additional information on cost of community health agent programs and the cost of scaling and sustaining such programs will support CRVS system strengthening efforts.

## Paper context

Previous literature has not explored factors integrating community health agents into civil registration and vital statistics (CRVS) strengthening efforts. In this review, a majority of articles cited household visitation by community health agents, though only one described vital events tracking activities being officially reported to a CRVS system. Creating a programmatic norm of linking vital events reported by health programs with CRVS systems could increase the coverage of officially reported vital events.
